# The variances of Sp1 and NF-κB elements correlate with the greater capacity of Chinese HIV-1 B′-LTR for driving gene expression

**DOI:** 10.1038/srep34532

**Published:** 2016-10-04

**Authors:** Di Qu, Chuan Li, Feng Sang, Qiang Li, Zhi-Qiang Jiang, Li-Ran Xu, Hui-Jun Guo, Chiyu Zhang, Jian-Hua Wang

**Affiliations:** 1 CAS Key Laboratory of Molecular Virology and Immunology, Institut Pasteur of Shanghai, Chinese Academy of Sciences, Shanghai, China; 2 Key laboratory of Prevention and Treatment with Traditional Chinese Medicine on Viral Infection Disease, the First Affiliated Hospital of Henan University of Traditional Chinese Medicine, Zhengzhou, China

## Abstract

The 5′ end of HIV-1 long terminal repeat (LTR) serves as a promoter that plays an essential role in driving viral gene transcription. Manipulation of HIV-1 LTR provides a potential therapeutic strategy for suppressing viral gene expression or excising integrated provirus. Subtype-specific genetic diversity in the LTR region has been observed. The minor variance of LTR, particularly in the transcription factor binding sites, can have a profound impact on its activity. However, the LTR profiles from major endemic Chinese subtypes are not well characterized. Here, by characterizing the sequences and functions of LTRs from endemic Chinese HIV-1 subtypes, we showed that nucleotide variances of Sp1 core promoter and NF-κB element are associated with varied LTR capacity for driving viral gene transcription. The greater responsiveness of Chinese HIV-1 B′-LTR for driving viral gene transcription upon stimulation is associated with an increased level of viral reactivation. Moreover, we demonstrated that the introduction of CRISPR/dead Cas9 targeting Sp1 or NF-κB element suppressed viral gene expression. Taken together, our study characterized LTRs from endemic HIV-1 subtypes in China and suggests a potential target for the suppression of viral gene expression and a novel strategy that facilitates the accomplishment of a functional cure.

HIV-1 long terminal repeat (LTR) promoter plays an essential role in driving viral transcription and productive infection^1^,^2^,^3^. The HIV-1 LTR is approximately 640 base pairs in length. It is segmented into the U3, R, and U5 regions. The U3 can be further subdivided according to transcription factor sites and the impact on LTR activity and viral gene expression. The enhancer regions usually consist of two highly conserved NF-κB binding sites, and the core region of LTR contains three tandem GC-rich binding sites for the Sp transcription factor family members, and the immediate TATA box for the target of TATA binding protein (TBP)^4^,^5^,^6^.

The sequence and function of LTRs from many HIV-1 subtypes have been well characterized. Subtype-specific genetic diversity in the LTR has been observed, and the subtle variance in LTR can have a significant impact on promoter/enhancer activity, replication kinetics and the virulence of different HIV-1 subtypes^2^,^7^,^8^,^9^,^10^,^11^,^12^. LTRs from HIV-1 B subtypes contain two NF-κB binding sites, whereas HIV-1 LTR from subtype C, which is the predominant clade in Africa, contains an additional NF-κB-like binding site and shows higher activity in activating viral gene transcription^13^,^14^,^15^. In contrast, CRF01_AE-LTR contains a single NF-κB site and the upstream NF-κB site usually presents in other subtypes is replaced by a GABP (GA binding protein) binding site. The NF-κB to GABP conversion in CRF01_AE-LTR might account for the increased level of basal transcription and the reduced response upon TNF-α stimulation, and thus may likely be a contributor to latent infection^9^,^10^,^16^. Additionally, CRF01_AE contains a different TBP binding site with the sequence TAAAA, whereas all other subtypes have a TATAA sequence; this single nucleotide variance reduces the efficiency of assembly of the TBP-TFIIB-TATA complex for the recruitment of RNA polymerase II^2^,^16^,^17^.

Notably, the subtle alterations in LTRs may affect disease progression^8^,^11^. An analysis in HIV-infected patients revealed that the C-to-T change at the Sp1 site III is positively correlated with disease progression^11^. The additional creation of a new NF-κB binding site and the insertion of a duplicating transcription factor site for RBEIII in LTRs of subtype C have been observed in therapeutic failure patients from Brazil and Mozambique; this may partially explain the greater fitness of subtype C and the substitution of subtype B in those regions^18^. This LTR sequence alteration may affect the modulation of viral latency. A systematic genetic screen for HIV LTR promoter elements revealed that single point mutations of the Sp1 site III at the 4^th^ guanine nucleotide and TATA box at the 2^nd^ adenine increased the frequency of switching from latency to productive HIV-1 infection^12^.

The indispensable roles of HIV-1 LTR in driving viral gene expression and maintenance of viral latency make it an attractive target for antiviral therapy. Several gene editing technologies, such as zinc finger nuclease (ZFN), transcription activator-like effector nuclease (TALEN) and clustered regularly interspaced short palindromic repeats (CRISPR)/CRISPR-associated (Cas) 9 (CRISPR/Cas9), have been used to remove the proviral HIV-1 DNA from the host cell genome by targeting its highly conserved LTRs^19^,^20^,^21^,^22^,^23^,^24^,^25^,^26^,^27^,^28^.

Previous studies have mainly characterized LTRs from HIV-1 B, C and AE subtypes that are more prevalent in Europe and America^2^,^29^, whereas the LTR and viral profiles from endemic Chinese HIV-1 subtypes have not been carefully characterized^30^. Several HIV-1 subtypes are co-circulating among various high-risk groups in China. Subtypes B′ (Thailand’s variant of subtype B) and CRF01_AE were introduced into China from Thailand, and subtype C was primarily transmitted from India^29^,^31^. CRF07_BC and CRF08_BC are two Chinese subtypes that most likely have originated from Yunnan province of China^32^. In the early stages of HIV epidemic, subtype B′ was mainly circulating among former plasma donors in Central China (mostly in Henan province), CRF01_AE was predominant among heterosexuals and injection drug users (IDUs), and CRF07_BC and CRF08_BC were prevalent among IDUs. In recent years, various HIV-1 subtypes have rapidly disseminated among different high-risk groups, an increased prevalence of CRF01_AE and CRF07_BC has been observed among men who have sex with men (MSM).

In this study, we cloned LTRs from Chines specific subtypes B′, CRF07/08_BC, and CRF01_AE, and compared their activities with those of global HIV-1 subtypes. We found that the HIV-1 B′-LTR sequence has the strongest capacity for driving viral gene expression. Sequence analysis revealed that, in addition to the variance in NF-κB binding sites, the variance in Sp1 core promoter determined its activity. Finally, we demonstrated that the Sp1 core promoter nucleotides and the NF-κB binding sites can serve as targets for CRISPR/Cas9 gene editing, and the depletion of these sites led to suppressed viral gene expression.

## Results

### The 5′-LTR from HIV-1 B′ subtype has the strongest capacity for driving gene expression

The 5′-LTR of HIV-1 has been shown to maintain subtype-specific genetic diversity^7^,^10^,^12^,^18^. To confirm this in prevalent HIV-1 subtypes in China, we downloaded 300 LTR sequences of circulating HIV-1 strains including B′ (54 sequences), CRF07_BC (42 sequences), CRF08_BC (36 sequences) and CRF01_AE (168 sequences) from the Los Alamos HIV database (http://www.hiv.lanl.gov/content/index, on April 21, 2014) (Table S1), and aligned these sequences to discern genetic variability. For comparison, LTR sequences from worldwide distributed subtype B (300 sequences) and subtype C (300 sequences) were from the same database and aligned by Mega software using Clustal W parameters. As expected, subtype-specific genetic diversity in the LTR was observed as shown in the phylogenetic tree analysis (Fig. S1). The 5′-LTR consensus sequences were highlighted (Fig. S2). CRF07_BC and CRF08_BC show mosaic patterns in a subtype C genomic backbone inserted by several subtype B′ fragments in *gag*, *pol* and *env* genes^33^,^34^. Because they have the same LTR consensus and are thus combined for the latter analysis. The LTR of B′ and B have two NF-κB binding sites; the LTR of CRF07/08_BC and CRF07/08_C have two NF-κB binding sites and one NF-κB binding-like site, respectively; the LTR of CRF01_AE has a single NF-κB enhancer. Besides the number of NF-κB binding sites, the other transcription factor elements such as TCF-1 (T-cell-specific transcription factor 1), Sp1 and TAR (Trans-activating region) also showed marked variations.

**Figure 1:**
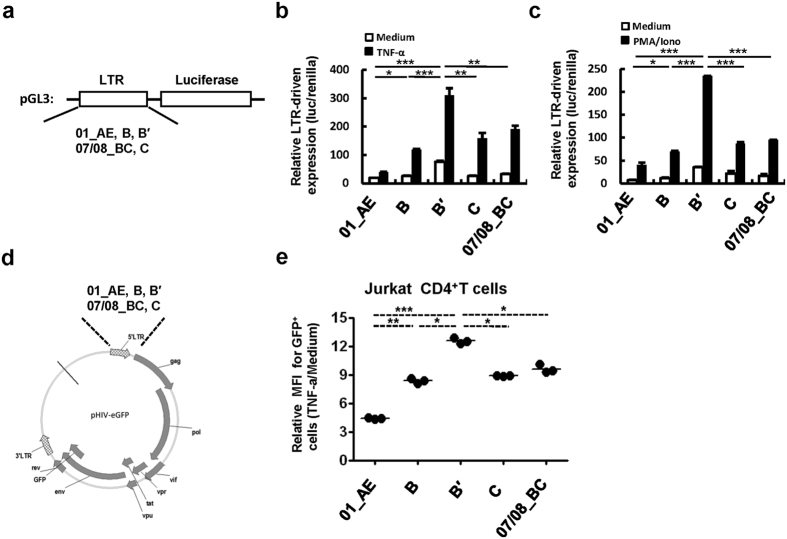
The 5′-LTR from HIV-1 B′ subtype demonstrates the strongest capacity for driving gene expression. (**a**) Schematic construction of pGL3 containing LTR-consensus-driven luciferase reporter. (**b**,**c**) Assay for LTR-driven gene expression upon stimulation. HIV-1 LTR-luciferase-reporter plasmids and pRenilla-luc-TK were co-transfected into HEK293T cells for 24 h and stimulated with TNF-α (**b**) or PMA/Ionomycin (**c**) for additional 24 h. The cells were harvested, and luciferase activity was measured and calculated by using dual luciferase reporter assay. (**d**) Schematic construction of pHIV-eGFP containing LTR consensus. (**e**) Infection of Jurkat CD4^+^ T cells. Jurkat CD4^+^ T cells were infected with pHIV-eGFP/VSV-G (1 ng p24^gag^ amount viruses for 10^6^ cells) for 24 h, with or without TNF-α (2 ng/ml) stimulation, and viral infection was determined by measuring GFP expression with flow cytometry, and the mean fluorescence intensity (MFI) was calculated. *P < 0.05, **P < 0.01 and ***P < 0.001 are considered significant difference in an unpaired t-test.

Previous studies showed that the minor variance in LTR can have a significant impact on its activity^2^,^8^,^9^,^11^,^12^. To investigate the potential difference in LTR activity from HIV-1 subtypes predominant in China, we synthesized these consensus LTR sequences and inserted them into a luciferase-based reporter vector pGL3 (Fig. 1a). These plasmids containing various LTR sequences were transfected into HEK293T cells and then the luciferase activities were measured. B′-LTR showed the strongest capacity for driving basal gene expression and elevated gene expression upon stimulation with either TNF-α or PMA/Ionomycin. In comparison, CRF07/08_BC-, C- and B-LTR displayed a moderate response to the same stimuli, whereas CRF01_AE-LTR exhibited the least activity under these experimental conditions (Fig. 1b,c).

To further investigate the activity of subtype-specific LTRs variants, we inserted synthesized LTR consensus nucleotides into pHIV-eGFP and replaced the 5′-LTR of this plasmid (Fig. 1d), and then co-transfected HEK293T cells with the expression plasmid for vesicular stomatitis virus G (VSV-G) protein. Culture supernatant containing the pseudotyped single-cycle infectious HIV-eGFP/VSV-G was used to infect Jurkat CD4^+^ T cells. The pseudotyped virus containing HIV-1 B′-LTR from Chines isolates showed the most activity for driving viral gene transcription upon stimulation with TNF-α (Fig. 1e). Taken together, these data confirmed the variation in the function of subtype-specific LTRs, and that the 5′-LTR from HIV-1 B′ has the strongest capacity for driving gene expression.

### The (-138)-0 fragment containing the enhancer/core promoter sequences determines the difference in LTR activity

**Figure 2:**
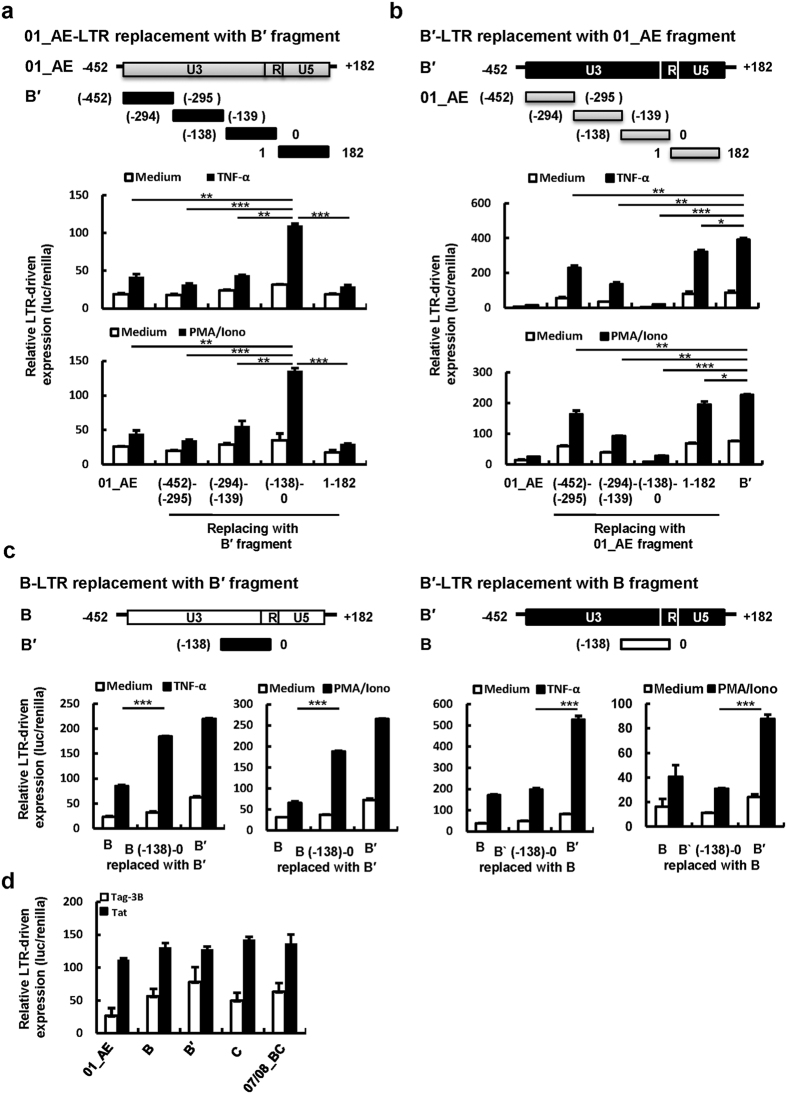
The (-138)-0 fragment determines the efficiency of LTR activity. (**a–c**) Assay for LTR-driven gene expression in transfected HEK293T cells upon TNF-α or PMA/Ionomycin stimulation. The homologous fragments were replaced as depicted in schematic graphs. Dual luciferase reporter system was used. (**e**) Investigation on Tat-driven *trans*-activation. HIV-1-*tat* expressing plasmid and plasmids containing various LTRs were co-transfected into HEK293T cells for 48 h and luciferase activities were measured. Data are mean ± SD. Results are representative of four independent experiments (**a–c**) and of three independent experiments (**d**). *P < 0.05, **P < 0.01 and ***P < 0.001 are considered significant difference in an unpaired t-test.

Multiple nucleotide alterations distributed throughout different transcription factor elements have been observed, and the most noted alterations are mainly found in the (-138)-0 fragment containing the enhancer/core promoter sequences (Fig. S2). To investigate whether variances in the (-138)-0 region determine the differences in LTR activity, we synthesized the (-138)-0 region of B′-LTR and used it to replace the homologous fragment in CRF01_AE-LTR. The (-452)-(-295) fragment, (-294)-(-139) fragment containing TCF-1 element variance and 1–182 fragment containing TAR variance were also synthesized for homologous replacement. The replacement with the (-138)-0 but not the (-452)-(-295), (-294)-(-139) or 1–182 region of B′-LTR significantly increased CRF01_AE-LTR activity upon TNF-α or PMA/Ionomycin stimulation (Fig. 2a). In contrast, when the (-138)-0 region of B′-LTR was replaced with homologous segment of CRF01_AE, B′-LTR significantly reduced its activity (Fig. 2b). Similarly, B-LTR acquired significantly enhanced activity after homologous introduction of B′-LTR (-138)-0 region (Fig. 2c). Conversely, B′-LTR significantly reduced its activity after the (-138)-0 region was homologously replaced with that of the B-LTR.

The 1–182 fragment contains TAR element (nt +1 to +59), which binds to HIV-1 Tat protein to allow transcription elongation. To further confirm that the 1–182 region of HIV-1 LTR is dispensable for the observed differences in LTR activity, we co-transfected HEK293T cells with HIV-1-*tat* expressing plasmid (pCMV-tat, cloned from HIV-1 NL4-3, B subtype) and plasmids containing various LTRs, to examine the variation in Tat-driven *trans*-activation. As expected, different LTRs showed similar response upon Tat activation (Fig. 2d), indicating that variation of LTR activity was not attributable to Tat-driven *trans*-activation and transcription elongation, and that the (-138)-0 region containing enhancer elements for transcription initiation was the key element responsible for the functional variability associated with LTR variations. Taken together, these data demonstrate that the (-138)-0 nucleotides in B′-LTR confer the variation in LTR′ ability for driving gene expression.

### The variances in Sp1 core promoter are associated with HIV-1 B′-LTR activity for driving gene expression

**Figure 3:**
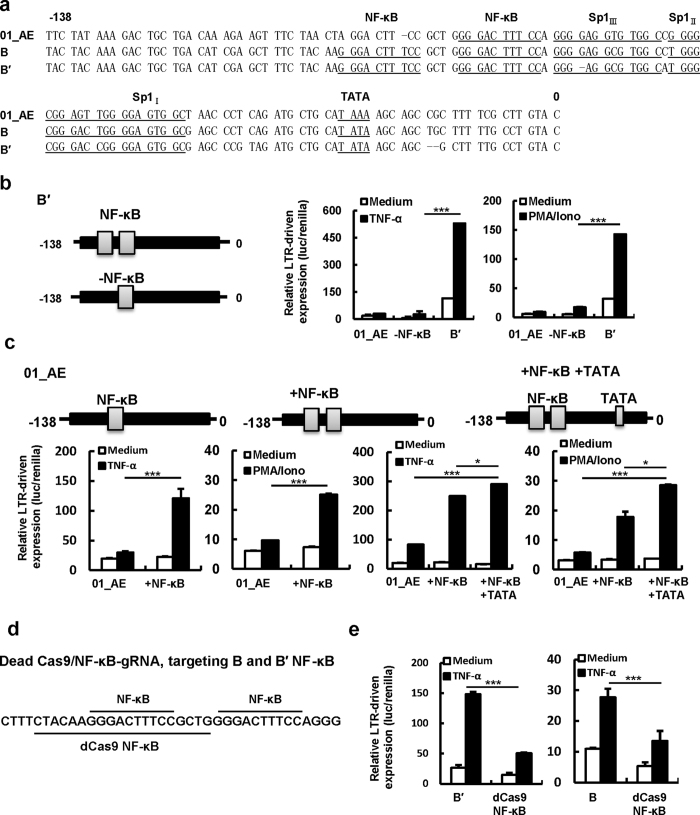
The frequency of NF-κB binding sites and the TATA regulatory element are essential for HIV-1-LTR activity. (**a**) Alignment of the core elements in (-138-0) fragment of CRF_01AE-, B- and B′-LTR. (**b**,**c**) Assay for LTR-driven gene expression. The first NF-κB binding site was depleted from B′-LTR (−NF-κB) (**b**). A NF-κB binding site was added (+NF-κB), or a NF-κB binding site and the TATA box were added (+NF-κB +TATA) to 01AE-LTR (**c**). (**d**) Design of a gRNA targeting the first NF-κB binding site of B′. (**e**) Assay for LTR-driven gene expression. HIV-LTR-specific dCas9/gRNAs and LTR-reporter plasmids were co-transfected into HEK293T cells, and cells were harvested for quantifying LTR activity after being stimulated with TNF-α for 24 h. Data are mean ± SD. Results are representative of four independent experiments. *P < 0.05, **P < 0.01 and ***P < 0.001 are considered significantly different.

We then concentrated on the (-138)-0 nucleotides of LTRs to investigate how its diversity affected gene expression using a reporter system. This (-138)-0 region contains multiple elements for transcription factor binding, including the NF-κB enhancer and Sp1 core promoter. The AE subtype contains only one NF-κB binding site, whereas B′ and B contain two (Fig. S2). Another apparent difference is that B′ and B each contains a TATA box in the core promoter, whereas CRF01_AE contains a TAAA sequence in this area (Fig. 3a). Indeed, the number of NF-κB binding sites influence subtype specific HIV-1 LTR activity, as the depletion of one NF-κB element from B′-LTR, or the insertion of an additional NF-κB element to CRF01_AE-LTR significantly altered LTR activity upon TNF-α or PMA/Ionomycin stimulation (Fig. 3b,c). To further prove the role of NF-κB binding sites on HIV-1 LTR capacity for driving viral gene expression, dCas9/gRNA system was used to silence NF-κB activation (Fig. 3d). We constructed NF-κB-gRNA first, and then co-transfected it with either B′- or B-LTR into HEK293T cells. As expected, the LTR activity was significantly reduced (Fig. 3e). Moreover, the addition of TATA box into CRF01_AE-LTR further enhanced LTR activity (Fig. 3c).

**Figure 4:**
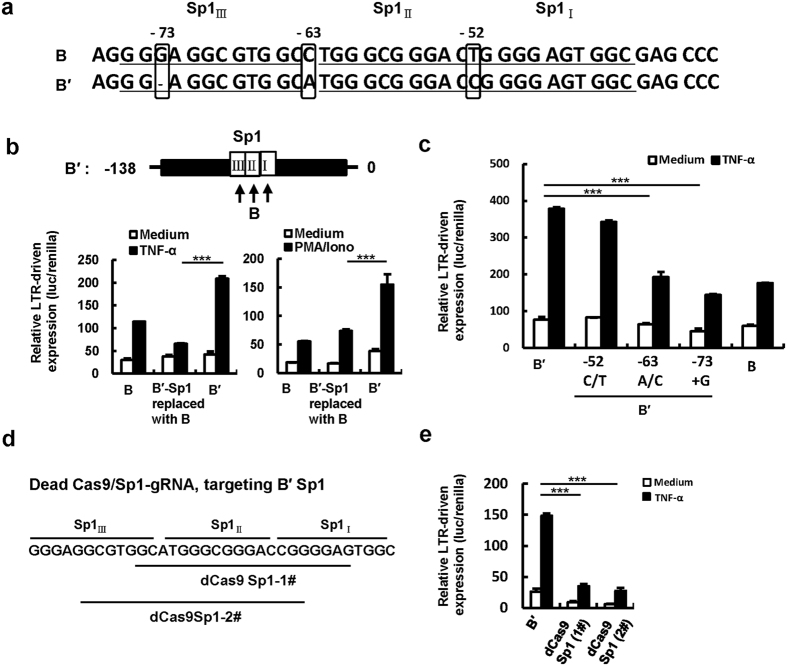
The variances in Sp1 element confer HIV-1 B′-LTR greater ability for driving gene expression and the introduction of CRISPR/dCas9 targeting Sp1 core elements suppressed viral gene expression. (**a**) Alignment of Sp1 element sequences. (**b**) The Sp1 elements of B′-LTR were replaced with B homologous fragments, and LTR activity was quantified after TNF-α or PMA/Ionomycin stimulation. (**c**) The varied bases in HIV- B′-LTR Sp1 element were mutated and the capacity for driving gene expression was measured. (**d**) Design of gRNAs targeting Sp1 transcription element of LTR. (**e**) Assay for LTR-driven gene expression. HIV-LTR-specific dCas9/gRNAs and LTR-reporter plasmids were co-transfected into HEK293T cells, and cells were harvested for quantifying LTR activity after stimulated with TNF-α for 24 h. Data are mean ± SD. Results are representative of three independent experiments. ***P < 0.001 are considered significant difference.

B′-LTR showed greater capacity for driving gene expression than B-LTR (Fig. 1b,c,e). Although B- and B′-LTR show high conservation in (-138)-0 regions (Fig. 3a), there are subtle variances between them in the Sp1 core promoter sequences. Compared with B′-LTR, cytosine is changed to adenine at −63 position, thymine is changed to cytosine at −52 position, and one guanine is depleted at −73 position in the Sp1_III_ and Sp1_II_ regions of B-LTR (Fig. 4a).

The replacements of B′-LTR (-138)-0 region or Sp1_III_ and Sp1_II_ elements with B homologous fragments significantly lowered its activity (Fig. 4b). To determine which base in the Sp1 element contributes to the stronger ability of B′-LTR, we mutated specific bases in B′-LTR, and found that the replacement of adenine at −63 position with cytosine and the adding of guanine at −73 position, but not the replacement of cytosine at −52 position with thymine, significantly impaired B′-LTR responses to stimulation (Fig. 4c), demonstrating the uniqueness of B′-LTR Sp1_III_ region is the key for enhanced activity. Taken together, these data prove that the variances in Sp1 elements contribute to the stronger capacity of HIV-1 B′-LTR to induce activation.

### The introduction of CRISPR/dCas9 targeting Sp1 core elements suppressed viral gene expression

The indispensable roles of HIV-1 LTR in driving viral gene expression make it an attractive target for designing new antiviral strategy. The CRISPR/Cas9 system is known to play a major role in adaptive immune responses against foreign pathogens in prokaryotes, and recently, it has been adopted to disrupt HIV-1 entry coreceptors C-C chemokine receptor 5 (CCR5), or excise HIV-1 provirus in order to suppress viral gene expression and eradicate viral latency^19^,^20^,^21^,^22^,^23^,^24^,^25^,^26^,^28^,^35^,^36^. To further prove the role of Sp1 core promoter on HIV-1 LTR driving viral gene expression in Chinese HIV-1 B′ subtype, dCas9/gRNA system was used to inactivate the activation of Sp1 transcription elements^28^. Two gRNAs were designed to target Sp1 transcription elements in LTRs (Fig. 4d). Transfection of these HIV-LTR-specific dCas9/gRNAs into HEK293T cells silenced HIV-1 B′-LTR-driven gene expression (Fig. 4e). Together, these data demonstrate that Sp1 core elements can be targeted by the CRISPR/Cas9 system for blocking HIV-1 gene expression.

### Virus containing HIV-1 B′-LTR shows the greatest responsiveness to experimental reactivation from latency

**Figure 5:**
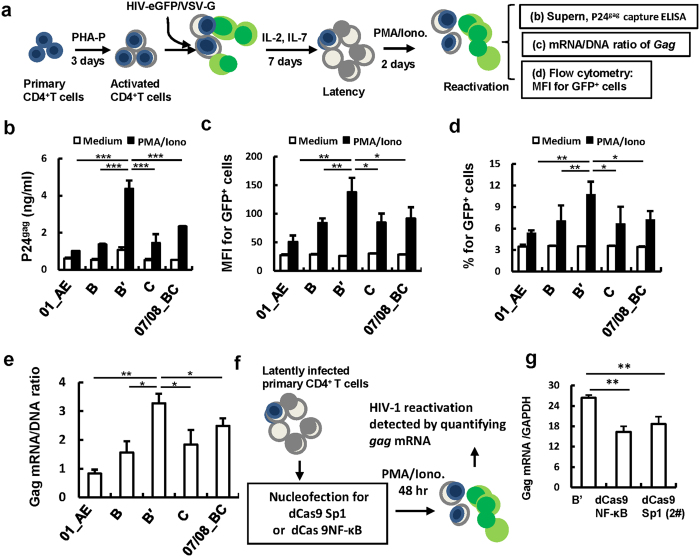
HIV-1 B′ virus shows greater capacity to induce reactivation from latency. (**a**) Establishment of HIV-1 latently infected primary CD4^+^ T cells. PHA-P-activated CD4^+^ T cells were infected with pseudotyped HIV-eGFP/VSV-G, and cells were cultured in the presence of IL-2 and IL-7 for 7 days. Viral latency was reactivated by PMA/Ionomycin stimulation, and newly synthesized viruses released in culture supernatant were detected with p24^gag^ capture ELISA (**b**). Viral reactivation from latency was also monitored by measuring GFP expression with flow cytometry (**c,d**), or quantifying the relative Gag mRNA/DNA ratio with quantitative (RT-) PCR (**e**). (**f**,**g**) The HIV-1 latently infected CD4^+^ T cells can be targeted by the Cas9/gRNAs system to suppress reactivation. dCas9-Sp1 or dCas9- NF-κB were nucleofected into HIV-1 latently infected primary CD4^+^ T cells (**f**), and viral reactivation from latency upon PMA/Ionomycin stimulation was quantified with quantitative (RT-) PCR to measure the Gag mRNA/DNA ratio (**g**).

Silencing HIV-1 LTR-driven transcription of proviral DNA is the key for maintaining HIV-1 post-integration latency, but the process is reversible^37^,^38^,^39^,^40^. To investigate whether LTR variation affects viral reactivation from latency, we used pseudotyped HIV-eGFP/VSV-G viruses containing variable synthesized LTR consensus nucleotides to infect activated primary CD4^+^ T cells to establish viral latency first, and then used PMA/Ionomycin to stimulate cells for viral reactivation (Fig. 5a). HIV-1 B′-LTR possessed the strongest capacity for driving viral reactivation from latency, as measured by either quantifying newly synthesized virions in culture supernatant or detecting Gag-GFP expression (Fig. 5b–d). And HIV-eGFP/VSV-G containing HIV-1 B′-LTR displayed the highest mRNA/DNA ratio in reactivated primary CD4^+^ T cells (Fig. 5e). Moreover, these LTRs in latent infected cells could be targeted by the dCas9/gRNAs system and resulted in a blockade of viral reactivation from latency (Fig. 5f,g). Together, these data demonstrate that HIV-1 B′-LTR possesses the strongest ability in reactivating viral transcription from latency, and the predominant LTR sequences that promote this activity may provide key targets for gene editing to achieve modulation of viral latency.

## Discussion

In this study, we characterized the sequences and functions of LTRs from major endemic HIV-1 subtypes in China, and discovered that Chinese HIV-1 B′-LTR consensus sequences possessed the strongest capacity for driving viral gene expression. Specifically, the nucleotide variances of Sp1 core promoter were associated with LTR capacity for driving viral gene transcription. Additionally, the higher responsiveness of B′-LTR to external stimulation determined the increased reactivation from latency. This study reveals a small number of nucleotides that have profound influence on LTR activity, and thus permitting the identification of specific target sites for manipulation to suppress viral gene expression.

LTR variances have been known to significantly impact on promoter/enhancer activity, viral replication kinetics, virulence and dissemination^2^,^8^,^9^. The mutation of Sp1 element in LTR, particularly the point mutation of Sp1 site III at the 4^th^ guanine, has been proven in multiple studies to significantly impair Tat-mediated transactivation^6^,^12^,^41^,^42^,^43^. Whether the same is true for HIV-1 subtypes prevalent in China has not been carefully studied. HIV-1 subtype B′ is mainly circulating among former plasma donors in Central China. Its Sp1 element includes two nucleotide mutations, which appeared to have conferred B′-LTR the greatest capacity for driving gene expression upon stimulation with TNF-α or PMA/Ionomycin. The increased basal transcription and enhanced sensitivity to TNF-α stimulation suggest that the B′ subtype is less likely to become latent. Also, B′-LTR’s strong activity and responsiveness to stimulation may confer this virus higher replication fitness in patients. Therefore, more attention should be paid to this subtype during HIV surveillance.

An increased prevalence of CRF01_AE was recently observed among MSM with more rapid disease progression. Multiple cellular and viral factors have been found to be correlated with rapid disease progression in CRF01_AE infection^44^. The NF-κB to GABP conversion in LTR in CRF01_AE confers this virus more fitness to out-compete other HIV subtypes in new infections^16^, although this conversion reduces viral responses to TNF-α-stimulation^10^. Whether the enhanced fitness is a direct result of the NF-κB to GABP conversion and a correlate of rapid disease progression in CRF01_AE infection are yet to be confirmed.

The evolution of LTR may be compartmentalized in tissues, and varied among individual patients. One study investigated the phylogenetic relationship among HIV-1 proviral LTR quasispecies existed in an AIDS patient’s postmortem tissues, including lymph node, spleen, lung, dorsal root ganglion and spinal cord, as well as PBMCs obtained 2 months prior death. The study found that the spinal cord and dorsal root ganglion harbored HIV-LTRs that were genetically distinct from that existed in other organs. Thus, LTR variance may reflect the adaptation to transcription factors in specific tissue environments^45^.

The indispensable roles of HIV-1 LTR in driving viral gene expression make it an attractive target for combating infection. The conserved regions in LTR have been targeted by gene editing techniques including ZFN, TALENS and CRISPR/Cas9 for editing or removing integrated proviral DNA to archive a functional cure^19^,^20^,^21^,^22^,^23^,^24^,^25^,^26^,^27^,^28^. We demonstrated that the Sp1 core element could be targeted by CRISPR/Cas9 targeting system for silencing gene expression. Our results are in agreement with a recent study demonstrating that the Sp1 binding sites could be targeted by the host restrictive factor tripartite motif-containing 22 for silencing HIV-1 transcription^46^.

Taken together, our study characterized the property of LTRs from major endemic HIV-1 subtypes in China, and confirmed the critical role of Sp1 and NF-κB elements for LTR activity. These findings permit the identification of potential target sites for manipulation to suppress viral gene expression.

## Methods

### Ethics statement

Peripheral blood mononuclear cells from health donors were purchased from Shanghai Blood Center, Shanghai, China. The usage of human cells, and the related methods and experimental protocols have been approved by the Medical Ethics Review Committee of Institut Pasteur of Shanghai, Chinese Academy of Sciences. All experiments were performed in accordance with relevant national guidelines and regulations.

### Cells

Peripheral blood mononuclear cells (PBMCs) were separated from buffy coats obtained from healthy donors using Ficoll-Paque density gradient centrifugation, and CD4^+^ T cells were further purified from PBMCs using anti-CD4 antibody-coated magnetic beads (Miltenyi Biotec). Resting CD4^+^ T cells were treated with 5 μg/ml phytohaemagglutinin-P (PHA-P) (Sigma) for 3 days in the presence of IL-2 (20 IU/ml) for activation. The HEK293T cells were kindly provided by Dr. Li Wu (The Ohio State University, USA). The cells were maintained in Dulbecco’s modified Eagle’s medium (Hyclone) containing 10% fetal bovine serum (Hyclone), 100 U/ml penicillin and 100 μg/ml streptomycin.

### Plasmid DNA

HIV-1 backbone-based vector pHIV-eGFP and vesicular stomatitis virus G (VSV-G) expression plasmids were gifts from Dr. Li Wu (The Ohio State University, USA). Plasmids containing B′-, B-, CRF07/08_BC-, C-LTR and CRF01_AE-LTR, were constructed by inserting synthesized consensus LTR sequences into the multiple-cloning site of luciferase-based reporter vector pGL3 (Promega). Recombinants and mutations were constructed based on LTR-luciferase-reporter plasmids using ClonExpress II One Step Cloning Kit and Mut Express II Fast Mutagenesis Kit (Vazyme). To insert synthesized LTR consensus nucleotides into pHIV-eGFP vector, two restriction enzyme sites (XbaI and ClaI) were constructed into pHIV-eGFP vector using Mut Express II Fast Mutagenesis Kit (Vazyme). Consensus LTR sequences were inserted into pHIV-eGFP vector through these two restriction enzyme sites.

### The construction of CRISPR/dCas9

The pX330 plasmid containing two expression cassettes, hSpCas9 and the chimeric guide RNA and Dead Cas9 plasmid were gifts from Dr. Huan-Zhang Zhu (Fudan University, China). The CRISPR/dCas9 system targeting transcription modulators has been previously used to regulate HIV-1 gene expression^47^,^48^. One target sequence for NF-κB binding site (5′-CTACAAGGGACTTTCCGCTG-3′) and two target sequences for the Sp1 transcription element (5′-GCATGGGCGGGACCGGGGAG-3′; 5′-GCGTGGCATGGGCGGGACCG-3′) were cloned into pX330 plasmids according to the genome engineering toolbox^49^. These target sequences were tested using the http://www.crispr.mit.edu tool to check for off-target effects. The scores showed that all of three designed targets had high quality guide. BLAST search against human whole genome confirmed the non-specific binding of these designed gRNAs to other host genes.

### Virus Stock

Lipofectamine 2000 (Invitrogen) transfection of HEK293T cells was used to generate virus stock according to the manufacturer’s instructions. Pseudotyped single-cycle pHIV-eGFP/VSV-G viruses were generated by co-transfection with different LTR-containing pHIV-eGFP vectors and VSV-G expression plasmids. Supernatants harvested from transfected cells containing viral particles were titrated with a p24^gag^ capture ELISA.

### Dual luciferase reporter assay

HEK293T cells were grown in a 24-well cell culture plate. Transfection was performed using Lipofectamine 2000 (Invitrogen) according to the manufacturer’s instructions. HIV-1 LTR-luciferase-reporter plasmids and pRenilla-luc-TK were used. At 6 hr post-transfection, medium containing a mixture of plasmids and transfection reagent was replaced with fresh DMEM supplemented with 10% FBS. At 24 h post-transfection, cells were treated with TNF-α (50 ng/ml) or PMA (20 nM)/Ionomycin (1.5 μM). The cells were harvested for luciferase activity after 24 hr of treatment. Briefly, cells were washed with phosphate buffered saline (PBS) and homogenized with 100 μl lysis buffer (Promega); lysate (10 μl) was mixed with luciferase assay buffer (10 μl) and the firefly luciferase activity was measured first on a Veritas luminometer (Turner BioSystems). The plate was then removed from the luminometer and mixed with Stop & Glo Reagent (10 μl) and Renilla luciferase activity was measured. Data recorded on the luminometer were analyzed.

### The establishment of viral latency in primary CD4^+^ T cells

PHA-P-activated primary CD4^+^ T cells (0.5 × 10^6^) were incubated with pHIV-eGFP/VSV-G (25 ng p24 ^gag^) for 5 h at 37 °C. Cells were further cultured for 7 days in the presence of IL-7 cytokine for the establishment of viral latency^50^,^51^. Viral reactivation was induced by PMA (20 nM)/Ionomycin (1.5 μM) stimulation and detected by analyzing Gag-GFP expression with a Forteassa flow cytometer (BD Pharmingen); or alternatively, HIV-1 *Gag* mRNA/DNA production quantified with Real Time-(RT-) PCR, or HIV-1 p24 production with a p24^gag^ capture ELISA.

### Real time-(RT-) PCR analysis

Total cellular RNA from infected cells was extracted with TRIzol reagent (Life Technologies) and then reverse transcribed to cDNA with ReverTra Ace qPCR RT Master Mix with gDNA Remover Kit (TOYOBO). Genome DNA (gDNA) from infected cells was extracted using QIAamp MiniElute DNA Kit (Qiagen) according to the manual. RT-PCR was performed using the Thunderbird SYBR qPCR Mix (TOYOBO) as described previously with an initial denaturation step for 10 min at 95 °C, amplification with 40 cycles of denaturation (95 °C, 30 s), annealing (55 °C, 30 s), and extension (72 °C, 30 s), followed by a final extension at 72 °C for 6 min. The primers used were as follows: GAPDH: forward, 5′-GGGAAATCGTGCGTGACAT-3′ and reverse, 5′-GTCAGGCAGCTCGTAGCTCTT-3′; Gag: Forward, 5′-GTGTGGAAAATCTCTAGCAGTGG-3′ and Reverse, 5′-CGCTCTCGCACCCATCTC-3′.

### Phylogenetic analysis

For phylogenetic analysis, the complete 5′-LTR nucleotide sequences of HIV-1 subtypes endemic in China and elsewhere in the world were obtained from the Los Alamos HIV database (http://www.hiv.lanl.gov/content/index, on April 21,2014) (Table S1). Sequence alignment was performed with the ClustalW program in the Molecular Evolutionary Genetics Analysis (MEGA) 5 software. A phylogenetic tree was constructed with the Neighbor-Joining method, using MEGA. The bootstrap test was performed with 500 replications.

### Statistics

Statistical analysis was performed using an unpaired t test with SigmaStat 2.0 (Systat Software, San Jose, CA, USA).

## Additional Information

**How to cite this article**: Qu, D. *et al*. The variances of Sp1 and NF-κB elements correlate with the greater capacity of Chinese HIV-1 B′-LTR for driving gene expression. *Sci. Rep.* **6**, 34532; doi: 10.1038/srep34532 (2016).

## Supplementary Material

Supplementary Information

Supplementary Table 1
