# Supplementary material for: The variances of Sp1 and NF-κB elements correlate with the greater capacity of Chinese HIV-1 B′-LTR for driving gene expression
Source: Sci Rep. 2016 Oct 4;6:34532. doi: 10.1038/srep34532 (PMC5048295; doi:10.1038/srep34532)
Supplement: Supplementary Information [file srep34532-s1.pdf]

The variances of Sp1 and NF-κB elements correlate with the greater capacity of Chinese HIV-1 B′-LTR for driving gene expression

Di Qu, Chuan Li, Feng Sang, Qiang Li, Zhi-Qiang Jiang, Li-Ran Xu, Hui-Jun Guo, Chiyu Zhang, Jian-Hua Wang

Figure legends

Figure S1. Phylogenetic tree of HIV-1 LTR sequences. Phylogenetic analysis was performed based on HIV-1 LTR nucleotide sequences from the HIV database. The phylogenetic tree was performed by the neighbor-joining method with MEGA version 5 software, and the reliabilities indicated at the branch nodes were evaluated using 500 bootstrap replications. Each isolate was specified with HIV database accession number, strain name, isolation place, isolation year, and sub genotype.

Figure S2. Alignment of HIV-1 LTR sequences. Different subtypes of HIV-1 LTR sequences were downloaded from the HIV database (http://www.hiv.lanl.gov/content/index) and aligned in the Mega software. Variations in elements for transcription factor binding were summarized.

Table S1. The information of HIV-1 subtypes endemic in China. 300 LTR sequences of circulating HIV-1 strains in China including B′ (54 sequences), CRF07_BC (42 sequences), CRF08_BC (36 sequences) and CRF01_AE (168 sequences) from the HIV database (http://www.hiv.lanl.gov/content/index, on April 21, 2014) were downloaded

Figure S1

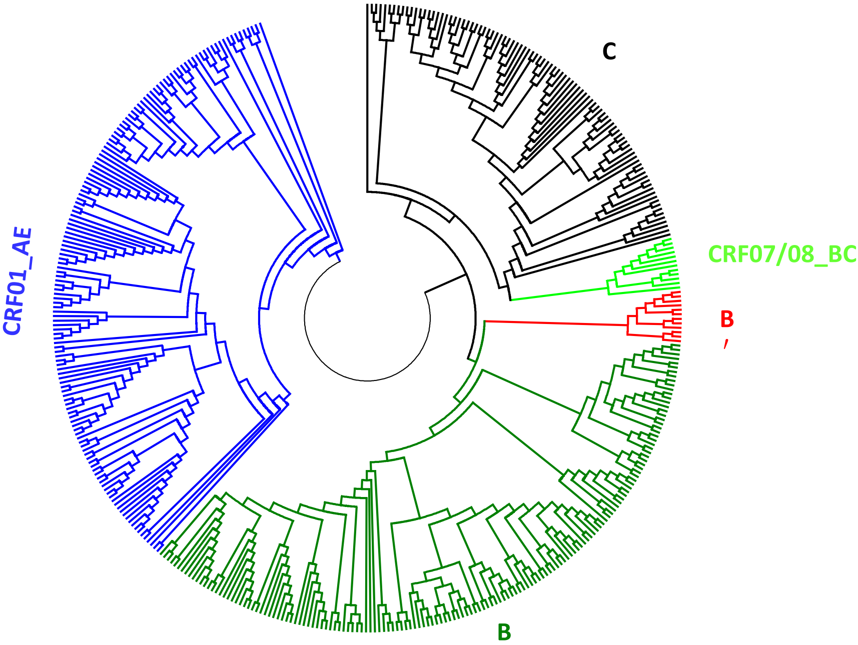

Figure S2

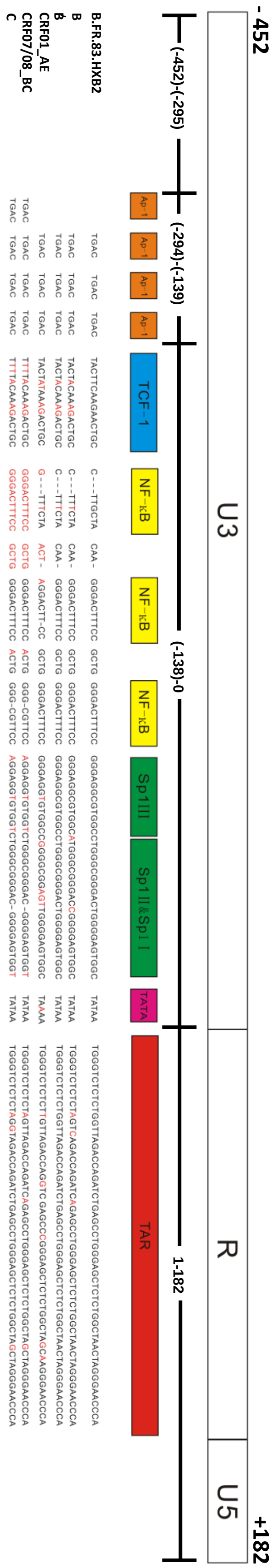

-452
+182
U3
R
U5
(-452)-(-295)
(-294)-(-139)
(-138)-0
1-182
Ap-1
TCF-1
NF-κB
Sp1 III
Sp1 II&Sp1 I
TATA
TAR
B.FR.83.HXB2
B
B'
CRF01_AE
CRF07/08_BC
C
